# The Ongoing Debate on the Use of Prophylactic Antibiotics in Acute Pancreatitis—Is There a Conclusion? A Comprehensive Narrative Review

**DOI:** 10.3390/antibiotics13050411

**Published:** 2024-04-30

**Authors:** Kai Siang Chan, Vishal G. Shelat

**Affiliations:** 1 Department of General Surgery, Tan Tock Seng Hospital, 11 Jalan Tan Tock Seng, Singapore 308433, Singapore; vgshelat@gmail.com; 2 Lee Kong Chian School of Medicine, Nanyang Technological University, Singapore 308232, Singapore; 3 Yong Loo Lin School of Medicine, National University of Singapore, Singapore 117597, Singapore

**Keywords:** antibiotics, cholangitis, culture, infection, microbiology, pancreatitis

## Abstract

Acute pancreatitis (AP) is a common but often self-limiting disease in the majority of patients. However, in the minority, who may progress to moderately severe or severe AP, high mortality risk has been reported. Infected pancreatitis necrosis (IPN) in necrotising pancreatitis has been shown to result in more than twice the mortality rate compared with in sterile pancreatic necrosis. This raises the question on whether prophylactic antibiotics (PABs) should be given in subgroups of AP to prevent superimposed infection to improve survival outcomes. Despite numerous randomised controlled trials (RCTs), meta-analyses, and guidelines on the management of AP, there is a lack of strong evidence to suggest the use of PABs in AP. Additionally, use of PABs is associated with antimicrobial resistance. Considerable heterogeneity exists and limits the interpretation of results—subgroup of AP benefitting from PAB use, choice/class of PAB, and timing of administration from symptom onset and duration of PAB use. Only a minority of existing meta-analyses suggest mortality benefits and reduction in IPN. The majority of existing guidelines do not recommend the use of PABs in AP. More research is required to make more definitive conclusions. Currently, PAB should only be administered after multidisciplinary discussions led by pancreatology experts.

## 1. Introduction

Acute pancreatitis (AP) is a commonly encountered pathology in general surgery and hepatopancreatobiliary surgery, with an approximate incidence of 50–80 cases per 100,000 people [1]. In the majority (80%) of cases, AP presents as a mild and self-limiting disease with a low mortality risk of 1–2% [2,3]. In mild AP, supportive management is provided with patient monitoring for risk of progression to severe AP, symptomatic relief, work-up of underlying aetiology, and administration of measures to reduce risk of recurrent AP [4]. However, for patients with moderately severe to severe AP (SAP), mortality risk has been reported to range from 20 to 40% [5,6,7]. Several international guidelines have been crafted to guide the management of SAP, aiming to reduce morbidity and mortality [8,9,10,11,12,13,14,15]. One big domain of contention is the role of prophylactic antibiotics (PABs) in SAP.

Recently published meta-analyses show conflicting results on the use of PABs. For instance, Ukai et al. demonstrated lower mortality for patients with acute necrotising pancreatitis (ANP) with PABs (administered within 72 h from symptom onset) [16], while Guo et al. showed similar mortality with and without prophylactic carbapenem use in SAP [17]. Several considerations need to be made, such as the presence of concomitant cholangitis, patient comorbidity, local resources, and available expertise. Despite the lack of concrete evidence supporting the use of PABs in AP, there is a considerable proportion of surgeons worldwide who administer PABs in AP [18]. This is causing growing global concern, especially with the silent epidemic of antimicrobial resistance (AMR), with an estimated 4.95 million deaths in 2019 [19]. It is necessary to rationalise the use of antibiotics and enforce governance to reduce the morbidity and mortality associated with AMR, calling for a timely update on the available literature on PABs. This review aims to summarise evidence on the role of PABs in AP with an up-to-date review of the available literature.

## 2. Definitions

To begin, there is a need to standardise the various terminologies used in the current literature. The indications for antibiotics can be classified into prophylactic, empirical, or therapeutic. Prophylactic refers to the administration of antibiotics to prevent an infection. Empirical antibiotics refer to administration to treat possible, probable, or suspected infection without definitive evidence that an infection is present, because the benefits outweigh the risks. Therapeutic refers to starting antibiotics with definitive proof that an infection exists. In the context of SAP, PABs are used when there is no evidence of an infection (e.g., afebrile, stable inflammatory markers, normal procalcitonin levels) and is used to prevent an infection, as this group of patients may deteriorate rapidly. Empirical antibiotics are used when there is suspected infection in SAP, such as concerns for infected pancreatic necrosis (IPN) and/or extra-pancreatic infections.

AP can be classified based on (1) severity, (2) morphological features, (3) types of local complications (if any), and (4) aetiology. The various definitions are summarised in Table 1. To date, the most commonly used system to stratify the severity of AP is the 2012 modified Atlanta classification system, where AP is stratified into mild, moderately severe, and severe [20]. There are other classification systems used in AP—though less commonly used—such as one proposed by the Pancreatitis Across Nations Clinical Research and Education Alliance (PANCREA) [21], where a four-tiered classification system (mild, moderate, severe, and critical) is used instead. Differences between that and the modified Atlanta classification system include the use of (peri)pancreatic necrosis instead of local complications, and classifying whether infection is present. In the classification system suggested by PANCREA, presence of infected (peri)pancreatic necrosis would place patients to be of at least severe AP, and critical AP would include both the presence of infected (peri)pancreatic necrosis (IPN) and persistent organ failure. One reason why presence of infection was used as a stratifying criterion would be the associated mortality. A meta-analysis by Werge et al. (n = 71 studies, 6970 patients) showed that IPN was associated with significantly higher mortality compared to sterile pancreatic necrosis (odds ratio (OR) 2.57, 95% confidence interval (CI): 2.00–3.31) [22]. This further reinforces the need to discuss the risks versus benefits of PABs in patients with sterile (peri)pancreatic necrosis.

It is also important to stratify AP based on morphological features and type of local complications due to the difference in prognosis and management [20]. Morphology of AP can be broadly classified into interstitial oedematous pancreatitis or necrotising pancreatitis. Pancreatic necrosis occurs because of impairment of pancreatic perfusion [20]. Local complications can be broadly classified into the following (diagnosed on imaging):

- Non-necrotic (sequelae of interstitial oedematous AP):
  - Acute peripancreatic fluid collection (APFC)—less than 4 weeks;
  - Pancreatic pseudocyst (absence of necrosis)—more than 4 weeks.
- Necrotic (sequelae of necrotising AP):
  - Acute necrotic collection (ANC)—less than 4 weeks;
  - Walled-off necrosis (WON)—more than 4 weeks.

In general, majority of patients with APFC are managed conservatively and these resolve without intervention [23]. While pancreatic pseudocysts are rare, these usually require some form of intervention, either endoscopically, surgically, or percutaneously [24]. In contrast, local complications of ANP are more worrisome, with risk of superimposed infection of ANC or WON—about 30% of patients with ANP develop IPN after the first week [25]. This is concerning, especially since the mortality of IPN is more than twice that of sterile necrosis [22].

## 3. Guidelines on AP

In view of the complexity of AP as well as the associated morbidity and mortality with SAP, numerous guidelines have been published over the past decades to guide clinicians on management. We have summarised the available guidelines on AP published since 2005 on their recommendations and quality of evidence regarding the role of PABs in AP without any infection (Table 2). Additionally, we also summarised the guidance on the use of biochemical markers and/or investigations to guide diagnosis of infection in AP, as well as the role and/or choice of antibiotics in AP with concomitant infection. Across all guidelines included in our review, none of them recommend PABs for all cases of AP regardless of severity. The majority also do not recommend PABs for SAP, except for the 2015 Japanese Guidelines, which state that PABs may improve prognosis in SAP if administered in the early phases of AP (<72 h from onset); however, this was a weak recommendation with a moderate level of evidence.

Nevertheless, despite the widespread availability of guidelines that do not recommend for PABs in AP, compliance is of concern. The MANCTRA-1 (coMpliAnce with evideNce-based cliniCal guidelines in the managemenT of acute biliaRy pancreAtitis) international audit from 2019 to 2020 showed that 83.4% and 53.4% of patients with severe acute biliary pancreatitis (ABP) and mild ABP, respectively, received PABs [27]. Other international surveys similarly supported this finding—163 of 1054 participants (15.5%) administered routine PABs for AP [18]. A plausible reason for the lack of compliance may be due to the clinicians’ fear of patient deterioration, especially in SAP where there is already organ dysfunction or lack of governance from stewardship teams. There is also a diagnostic dilemma on determining the presence of an infection in AP. The above reasons are drivers of AMR, which was described early on, such as in the 2006 guidelines by the American College of Gastroenterology [14]. However, subsequent guidelines do not cover the issue about the emergence of AMR and impact on outcomes, which highlights a concerning lack of awareness of the high mortality that ensues from AMR.

It is our view that pancreatologists are direct stakeholders of patient care and retain the stewardship of antimicrobial needs of their patients and work in tandem with institutional stakeholders to make joint decisions for individual patients. One needs to maintain a balance of antibiotic overuse, misuse, and abuse, and avoid that a deserving patient comes to harm from underuse, delayed use, or non-use. Frivolous use of antibiotics also results in wastage and environmental harm. Pancreatologists may also initiate somatostatin analogues to reduce pancreatic secretion. However, there is a role for multidisciplinary management of patients with AP given the clinical conundrums that exist; pancreatologists should work in tandem with other subspecialty experts—intensivists and radiologists. Patients with SAP have single- or multi-organ failure, which necessitates higher level acuity care, led by intensivists. Holistic management and supportive treatment of every organ system by intensivists are crucial. Referral to gastroenterologists for endoscopic retrograde cholangiopancreatogram (ERCP) should be considered when cholelithiasis or choledocholithiasis is the aetiology (which may also be iatrogenic from the use of somatostatin analogues) [28], but subject to the haemodynamic stability of the patient. Radiologists should be approached in the presence of intra-abdominal collections or suspicion of IPN. Confirmation of IPN requires culture confirmation by needle aspiration, which is invasive and has its risks. Hence, we will discuss the current literature on PABs in AP.

## 4. The Current Literature on the Role of Prophylactic Antibiotics in Acute Pancreatitis

Over the past decade, there have been several meta-analyses performed to try to pool results and identify whether PABs improve outcomes in AP. We have summarised the available meta-analyses (n = 8) in the literature published from 2010 to 2023 with the inclusion criteria (type of study, severity of AP), type and time of PAB administration, as well as a summary of effect size for each outcome variable obtained from the pooled studies in Table 3. Of these meta-analyses, there were six (75.0%) that included only RCTs [16,29,30,31,32,33], and six (75.0%) that included only SAP and/or ANP [16,17,31,32,33,34]. Table 4 summarises the number of meta-analyses which reported the outcomes of interest and have been stratified based on their study type and severity of AP.

Of the three studies that reported on overall infection rates, two reported a significant reduction in overall infection with PABs use [17,29]. Only a minority of meta-analyses demonstrated a statistically significant reduction in mortality (n = 2/8) [16,34], IPN (n = 2/8) [16,32], or extrapancreatic infections (EPIs) (n = 1/7) [30]. No studies showed a significant reduction in incidence of pneumonia (n = 3), need for surgical intervention (n = 7), or organ failure (n = 2). Of the included meta-analyses, only the review by Villatoro et al. and Lim et al. collected data on AMR from the included original articles [33,34], of which, only two original articles reported data on AMR [35,36].

There are a few reasons for the heterogeneity in results, and considerations need to be made:

- Morphological features and severity of AP;
- Choice/class of PABs;
- Timing of administration of PABs from symptom onset;
- Duration of PABs use.

### 4.1. Morphological Features and Severity of AP

The inclusion criteria of the various meta-analyses differ; some included studies with AP of any severity, while some only included SAP and/or ANP. Both SAP and ANP are associated with mortality risks of up to 30% [26,37]. As described above, IPN is associated with more than twice the mortality rate compared with sterile necrosis [22]. Hence, results from studies on SAP and/or ANP alone are likely to be different compared to those that included any severity of AP. Only the reviews on severe AP and/or ANP showed a significant reduction in mortality and IPN with PABs use (Table 4). This reinforces the hypothesis that since patients with SAP and/or ANP are in a pro-inflammatory state with high risk of deterioration [38], PABs may help to reduce the risk of superimposed infection and resultant mortality. In patients with SAP and/or ANP, gastrointestinal mucosal hypoperfusion can disrupt the mucosal defence barrier and thus allow for portal venous access to the enteric microbial flora. This hypothesis is supported by Garret et al., who showed that presence of 1–2 system and ≥3 system organ failure was associated with 4.44 times and 28.67 times the odds of IPN respectively compared with no organ failure [39]. Hence, PABs may have some role in selected SAP and/or ANP patients to reduce risk of IPN and subsequent mortality.

### 4.2. Choice/Class of PABs

On top of the controversy behind the use of antibiotics, there is also a need to consider the class of antibiotics used, as the pharmacokinetics of each class of antibiotics differ. Not all classes of antibiotics are able to penetrate pancreatic parenchyma, hence appropriate class of antibiotics should be chosen [10]. The pathogens responsible for IPN are usually Gram-negative bacteria (e.g., *Escherichia coli*, *Klebsiella pneumoniae*, *Proteus* spp.) due to bacterial translocation from gastrointestinal flora [40]. However, Gram-positive bacteria such as *Staphylococcus aureus*, *Streptococcus faecalis*, and *Enterococcus* spp. are also not uncommon, with reported incidence of about 30% in those with positive fluid cultures [41]. Clinicians should ensure that PABs, if used, should cover both Gram-negative and Gram-positive bacteria.

The majority of the meta-analyses performed thus far did not limit their inclusion criteria on the choice of PABs used, except for the study by Guo et al. in 2022, which analysed seven studies (five RCTs and two retrospective studies) with 3846 patients who received prophylactic carbapenem for SAP within 48–120 h from the onset of symptoms [17]. They showed a reduction in incidence of overall infections and overall local and/or systemic complications, but no statistically significant differences were observed in mortality, IPN, or EPI (Table 3). Carbapenems are one of the broadest antimicrobials against Gram-negative organisms and Gram-positive organisms, except for methicillin-resistant *Staphylococcus aureus* (MRSA) and *Enterococcus faecium*. The World Society of Emergency Surgery (WSES) 2019 guidelines suggest the use of broad-spectrum antibiotics in order to penetrate pancreatic necrosis and cover against both Gram-negative and Gram-positive microbes (strong agreement, moderate quality of evidence) [10]. Similarly, the AGA clinical practice update on the management of pancreatic necrosis recommends the use of broad-spectrum antibiotics such as carbapenems, quinolones, and metronidazole [26]. However, these recommendations were for the use of empiric antibiotics against suspected/proven IPN, but not for the use of PABs to prevent IPN/other infective complications in AP.

Yao et al. did not show a significant difference in mortality between PABs vs. no PABs in RCTs on ANP [32]; however, a subgroup analysis performed based on the class of antibiotics showed that prophylactic cephalosporins were associated with a significant reduction in mortality (RR: 0.71, 95% CI: 0.43–1.18), but not for beta-lactams in general (RR: 0.81, 95% CI: 0.47–1.38). One possible consideration is that beta-lactams include the use of broad-spectrum carbapenems; PABs with carbapenems raise concerns of increasing incidence of carbapenem-resistant bacteria, with a reported incidence of 33% accounting for EPI [42]. The use of carbapenems has also been postulated to result in a change in intestinal flora towards a higher incidence of Gram-positive bacteria [43], and may increase AMR. This is concerning, especially when 63% of patients with suspected/proven pancreatic infections had multi-drug-resistant (MDR) organisms [44]. Therefore, there is a need to balance the extent of antimicrobial coverage versus the risk of emerging AMR.

### 4.3. Timing of Administration of PABs from Symptom Onset, and Total Duration of PABs

In the management of sepsis, early antibiotic administration, during the “golden hour”, is prudent [45]. Reducing time to first antibiotics from more than 6 h to less than 1 h has been shown to reduce mortality by 9.5% [46]. Similarly, PABs for surgical procedures should be administered 30 to 60 min prior to surgical incision [47]. The evidence for this was based on antibiotic pharmacokinetics to achieve adequate plasma concentrations [48]. For PABs in AP, various timings have been used in the available literature, ranging from 48 to 120 h from symptom onset (Table 2). However, several of the included RCTs did not specify the timing of PABs administration [49,50]. The meta-analysis by Ukai et al. was the only one that had strict inclusion criteria of PABs administration within 72 h from symptom onset, or 48 h after admission; they showed lower mortality and IPN in the PABs group [16]. Lack of statistical significance in other meta-analyses may be due to the lack of standardisation of timing of antibiotics administration. However, it is apparent that several existing meta-analyses do not take into account the clinical significance of the timing of PABs administration, as this was not included in any subgroup analyses or their reported data [29,31,32,33].

Similarly, duration of PABs is another controversial issue. Antibiotic stewardship is an evolving concern due to over-prescription of antibiotics with risk of adverse events and AMR. Recommendations are made for a limit of 5–7 days in established intra-abdominal infection with no source control procedure [51]. Specific pathologies have been shown to require a protracted course of antibiotics, such as in cholecystitis and cholangitis complicated by bacteraemia [52], or pyogenic liver abscess (PLA). In the case of PLA, antibiotics are administered for a minimum of 2 weeks, with total duration of antibiotics guided by clinical and radiological response [53]. However, these are cases with a proven infective source. In the case of PAB use in AP, the duration of use varied from 5 to 21 days [35,36,54]. A protracted course of PABs may not necessarily be better at preventing infections, and vice versa. A 24 h course of PABs in elective plastic surgery has been shown to be as efficacious as a 5-day course in reducing surgical site infections [55]. Additionally, with such varied duration in PABs use, interpretation is difficult, and standardisation should be performed in subsequent studies. Additionally, we suggest that the duration of PABs be guided by clinical judgement and serologic biomarkers to avoid the risk of adverse events and AMR from a protracted course of antibiotics.

### 4.4. Antimicrobial Resistance

The emergence of AMR and its associated mortality are extremely concerning, especially in SAP, where mortality is already high. A global study using predictive statistical modelling also showed that in 2019 alone, *E. coli* and *K. pneumoniae—*common pathogens involved in gastrointestinal infections, including IPN—were each responsible for more than 250,000 deaths associated with AMR [19]. Lee et al. reported a high incidence (63%) of MDR organisms with suspected or proven pancreatic infections. Patients with MDR-associated infections had longer intensive care unit (ICU) stays (20 days vs. 2 days, *p* = 0.001) compared to those with non-MDR infections [44]. Although they showed that mortality was not statistically significant between MDR vs. non-MDR pancreatic infections (14% vs. 6%, *p* = 0.411), an absolute difference of 8% is clinically significant, and a lack of statistical significance may be due to lack of power.

Of the included reviews, only two original articles reported on AMR. Isenmann et al. reported a significant increase (*p* < 0.0001) in ciprofloxacin-resistant organisms in the PABs group (received ciprofloxacin) [35], and Dellinger et al. reported incidence of five bacterial isolates resistant to meropenem in each group [36]. However, the studies did not report which organisms were cultured. Ideally, studies evaluating the role of PABs should include microbiology results and culture sensitivities in patients who subsequently develop IPN or EPI to identify whether the use of PABs increases the incidence of MDR organisms. Subgroup analysis should also be performed to compare mortality of PABs vs. no PABs in patients with IPN and EPI. If sufficient well-designed studies demonstrate an increase in AMR without any reduction in mortality, IPN, EPI, and/or other improvements in study outcomes, this may call for the termination of further studies due to increased risks of harm to the PABs group.

### 4.5. Local Institutional Practice and Overall Management of Severe Acute Pancreatitis

Locally, in our institution, PABs are not routinely used for patients with SAP or ANP. If required, empirical antibiotics (and not prophylactic) are used for patients with AP and concomitant cholangitis or other EPIs (e.g., pneumonia or UTI). This is justified due to overlapping symptoms, clinical signs, and serologic biomarkers for both acute cholangitis and AP. The only distinction would be blood culture result, which takes 48–72 h, and a patient with suspected cholangitis should not be deprived of antibiotics. Thus, based on our local antibiogram, intravenous amoxicillin–clavulanate is used with a single dose of gentamicin for patients with suspected cholangitis.

For EPI, the choice of empiric antibiotics is based on its source—such as the use of piperacillin–tazobactam with a single dose of vancomycin to cover for hospital-acquired pneumonia. However, should the patient present with fever and/or haemodynamic instability with no localising source, it is up to the clinician to exercise clinical acumen to decide on an appropriate class of antibiotics. In this context, common choice of empiric antibiotics would be meropenem due to its broad-spectrum coverage. However, we also recommend multidisciplinary input. It is essential that the decision is led by clinical pancreatology experts who retain the direct oversight of clinical management. The choice and duration are based on local antibiogram and policies and allow for some subjectivity in clinical decision-making within the governance of local audits that form the local protocols. It is advocated that antibiotics are not a panacea to treat febrile episodes nor magic bullets to bring down elevated inflammatory markers. Lastly, a distinction is made in the ethos of prophylaxis versus therapy. A patient who has a documented infection rightfully deserves antibiotics to improve survival chances and should not be deprived of them. Often, it is witnessed that a patient does not have a documented infection, but the pancreatologist suspects infection based on their clinical wisdom, and this causes a dilemma on two fronts: (a) whether to observe or initiate antibiotics, and (b) if initiated, whether the antibiotic would be labelled as prophylactic or therapeutic. It remains our policy that when a pancreatologist suspects an infection, he/she retains the authority to initiate antibiotics, and such practice should not be claimed as prophylaxis due to its therapeutic intent. It is strongly encouraged to review the antibiotics with aggressive attempts for microbial isolation or consider withdrawal of antibiotics, again guided by the clinical course.

**Figure 1:**
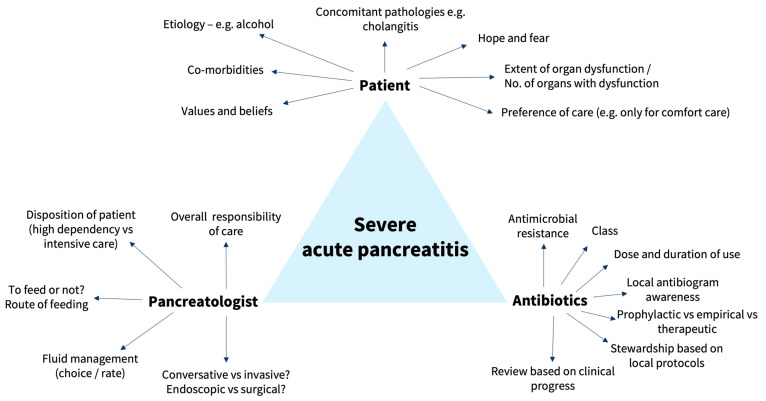
Important triad and associated factors in the management of severe acute pancreatitis.

The management of SAP is complex. Chan et al. previously summarised the various controversies in the management of AP—extent of work-up for establishing aetiology of AP, comparison between various risk stratification scores, guidance on choice and amount of fluid resuscitation, indications for ICU admission, mode of nutrition, role of ERCP for gallstone pancreatitis, and indications for invasive interventions in SAP [4]. Fluid resuscitation is critical to restore microcirculation to the pancreas, which has been regarded as the most important pathophysiologic goal for ischemic AP [56]. However, caution is to be taken to avoid excessive fluid replacement, which can cause other complications such as dilutional coagulopathy and reperfusion-mediated injury [8]. Oral and/or enteral feeding should be used (if possible) to reduce the risk of intestinal mucosal atrophy, maintain the intestinal barrier, and prevent bacterial translocation from the gut into the pancreatic necrosis, which can result in IPN and sepsis [57,58]. However, in the event of IPN, drainage and/or debridement of pancreatic necrosis is indicated but should be attempted using a step-up approach via endoscopy or minimally invasive surgery [10,26]. Pancreatic debridement should be attempted at least 4 weeks from the initial diagnosis of SAP in view of the increased morbidity and mortality associated with pancreatic debridement in the acute period [26]. Delayed drainage in IPN was not inferior to immediate drainage; in addition, patients with delayed drainage had fewer invasive interventions compared with the immediate drainage group [59]. We have summarised the important triad and considerations to guide the management of SAP in Figure 1.

### 4.6. Should More Studies Be Conducted, or Is Existing Evidence Sufficient to Prove a Lack of Benefit?

Most meta-analyses have thus far failed to show a mortality benefit (only n = 2/8 meta-analysis showed reduction in mortality [16,34]) and reduction in IPN (only n = 2/8 showed reduction in IPN [16,32]). This raises the question on how many more studies are required before a conclusion can be definitively made on the use of PABs for AP. One flaw of meta-analyses is that repetitive testing of significance from pooling of results may lead to an overestimation of results when data are sparse, leading to false positives (type 1 error) or false negatives (type 2 error). Trial sequential analysis (TSA) allows for controlling for random errors [60]. Poropat et al. performed a meta-analysis in 2022 on 21 RCTs (PAB n = 703, no PAB n = 680) and concluded that PABs do not reduce mortality or IPN [29]; however, incidence of sepsis, UTI, and LOS were lower in the PAB group. The authors additionally performed TSA and showed that the Z-curve did not cross the constructed monitoring boundaries (i.e., insufficient sample size to conclude that there is indeed reduced sepsis and UTI), and also did not cross the futility boundaries (i.e., insufficient sample size to conclude that mortality is not affected by PABs). With an alpha of 5% and beta of 20%, and an estimated relative risk reduction of 30%, a total of 2714 patients were required (only 1076 patients were included) for mortality (estimated incidence of 10%), and 1383 patients were required (only 512 patients were included) for UTI (estimated incidence of 20%). Hence, further RCTs and subsequent TSA should be conducted to determine if there is indeed a lack of benefit of PABs in AP.

## 5. Use of Serologic Biomarkers

As AP results in systemic inflammation and release of cytokines, it is therefore not surprising that inflammatory markers such as total white count (TWC) and C-reactive protein (CRP) are raised. Several scoring systems such as the Ranson score, Glasgow–Imrie score and APACHE II score use TWC as a surrogate marker of severity of inflammation i.e., severe AP [4]. Superimposed infection, which may be a sequelae of AP, similarly results in raised inflammatory markers, creating a conundrum on whether raised inflammatory markers are due to worsening inflammation versus a superimposed infection. In our opinion, trends of the markers when reviewed by pancreatology experts are paramount to informing clinical decision-making. We advocate pancreatology experts to retain autonomy to make decisions about antibiotics, as they follow the patients’ daily progress and are directly relevant by virtue of their ability to read and interpret computerised tomography scan images, distinct from non-pancreatology teams, who more often derive information by reading radiology reports or sporadically visit patients and provide episodic inputs.

Procalcitonin (PCT) is an increasingly used biomarker to differentiate between infective and non-infective causes of inflammation with higher specificity and sensitivity [61]. Studies have also been conducted to evaluate its use in severity stratification, such as in acute cholecystitis [62]. However, results have been inconclusive. The PROCAP trial, comparing the use of PCT-guided care vs. standard care, showed that serial trending of PCT was associated with reduced antibiotic use (45% vs. 63%, adjusted risk difference: −15.6%, *p* = 0.0071) without increasing the incidence of clinical infections or hospital-acquired infections in AP patients [63]. We suggest that PCT be used when faced with a conundrum on whether an infection is suspected, alongside the clinical progress and other clinical parameters of the patient.

Other biochemical markers that have been used to distinguish between IPN vs. sterile necrosis include phospholipase A_2_ (PLA2) [64], cortisol-binding globulin (CBG) [65], soluble triggering receptor expressed on myeloid cells (sTREM1) [66], and interleukin-6 (IL-6) [67]. However, these biochemical markers are not routinely measured clinically due to accessibility, affordability, availability, and lack of strong evidence to justify economics. Even if evidence does show that these biomarkers are able to predict risk of infection in AP, there is a need to improve their assay methods and lower costs for them to be valuable in clinical practice [68].

## 6. Role of Prophylactic Anti-Fungals in Acute Pancreatitis

Use of prophylactic anti-fungals is even more controversial in the management of AP. Translocation of gut bacteria has been postulated to be the pathogenesis of AP [40]. However, the gut microbiome also consists of fungi and it is therefore possible that both bacteria and fungi may translocate and colonise the pancreas, resulting in development of secondary infection [69]. Werge et al. and Rasch et al. reported that about 40% of patients with SAP and WON had fungal infection [70,71]. Rasch et al. also reported higher in-hospital mortality in patients with *Candida* spp.-positive fluid cultures compared to *Candida* spp.-negative fluid cultures (35.2% (n = 19/54) vs. 13.4% (n = 11/82), *p* = 0.003) [71].

Given the high incidence of fungal infections with associated increased mortality in AP, this raises the question as to whether prophylactic anti-fungals should be given to all patients, a select group of critically ill patients (e.g., SAP), or patients at risk of deterioration. For instance, female sex (OR: 3.13, 95% CI: 1.28–7.69) and post-ERCP pancreatitis (OR: 4.32, 95% CI: 1.01–18.36) were associated with increased risk of *Candida*-superimposed infections in ANP [71]. Additionally, broad-spectrum PABs may lead to the development of invasive pancreatic candidiasis due to stimulation of bacterial and *Candida* overgrowth [72,73]. However, to date, while there have been studies showing that prophylactic anti-fungals may reduce the risk of fungal infections in SAP, no RCTs have been performed to compare anti-fungal vs. control in preventing fungal infections in AP. The AGA clinical practice update also does not recommend the routine use of anti-fungals even when IPN is suspected [26]. Based on the above review, we believe that routine empirical anti-fungal prophylaxis should not be administered in AP and should only be administered in the presence of strongly suspected or documented fungal infection/fungaemia.

## 7. Conclusions

While SAP only occurs in a minority of patients, it bears high morbidity and mortality. Therapeutic antibiotics provide mortality benefit in patients with IPN, or AP with concomitant cholangitis. However, the role of PABs requires more studies to show a benefit. At present, a clinical conundrum exists; in view of the lack of evidence, choice of PABs should be guided by clinical progress, serologic biomarkers, and multidisciplinary discussions led by pancreatology experts who audit local protocols with organisational governance. Clinicians should also be cognizant of the emergence of AMR and need to rationalise antibiotic administration. On the contrary, there is no role for routine anti-fungal prophylaxis.

## Figures and Tables

**Table 1:** Summary of the various definitions used in acute pancreatitis.

Stratification	Name	Description	Imaging Findings on CECT
Severity	2012 modified Atlanta classification system [20]	Three-tier classification system: Mild—no organ failure, no local or systemic complications. Moderately severe—organ failure that resolves within 48 h, and/or local or systemic complications without persistent organ failure. Severe—persistent organ failure > 48 h.	N/A
	PANCREA classification system [21]	Four-tier classification system: Mild—absence of both (peri)pancreatic necrosis and organ failure. Moderate—presence of sterile (peri)pancreatic necrosis and/or transient organ failure. Severe—presence of either infected (peri)pancreatic necrosis or persistent organ failure. Critical—presence of both infected (peri)pancreatic necrosis and persistent organ failure.	N/A
Morphological features * [20]	Interstitial oedematous pancreatitis	Diffuse (or occasionally localised) enlargement of pancreas due to inflammatory oedema.	Pancreatic parenchyma enhancement by intravenous contrast, and No findings of peripancreatic necrosis
	Necrotising pancreatitis	Necrosis of pancreatic parenchyma, peripancreatic tissue, or both.	Lack of pancreatic parenchymal enhancement by intravenous contrast agent, and/or Presence of findings of peripancreatic necrosis
Local complications * [20]	Acute peripancreatic fluid collection (APFC)	Peripancreatic fluid with interstitial oedematous pancreatitis. No associated peripancreatic necrosis. No features of pseudocyst. Within first 4 weeks of interstitial oedematous pancreatitis.	Homogenous collection with fluid density Confined by normal peripancreatic fascial planes No definable wall encapsulating the collection No intrapancreatic extension
	Pancreatic pseudocyst	Encapsulated collection of fluid with well-defined inflammatory wall. Minimal or no necrosis. More than 4 weeks after onset of interstitial oedematous pancreatitis.	No non-liquid component Well defined and completely encapsulated
	Acute necrotic collection (ANC)	Collection with both fluid and necrosis. Associated with necrotising pancreatitis.	Heterogenous and non-liquid density of varying degrees in different locations No definable wall encapsulating the collection Can be intrapancreatic and/or extrapancreatic
	Walled-off necrosis (WON)	Encapsulated collection of pancreatic and/or peripancreatic necrosis that has developed within a well-defined inflammatory wall. More than 4 weeks after onset of necrotising pancreatitis.	Heterogenous and non-liquid density of varying degrees of loculations Well defined and completely encapsulated Can be intrapancreatic and/or extrapancreatic

* Definitions are obtained based on the 2012 modified Atlanta classification system. CECT: contrast-enhanced computed tomography; N/A: not applicable; PANCREA: Pancreatitis Across Nations Clinical Research and Education Alliance.

**Table 2:** Summary of existing guidelines on the role of antibiotics in acute pancreatitis.

Year	Professional Organisation/Association	Recommendation	Grade of Recommendation	Level of Evidence
2022	Korean Pancreatobiliary Association ^a,b^ [9]	Routine use of PABs not recommended in AP.	Strong	High ^b^
2022	French Society of Anaesthesia and Intensive Care Medicine, French National Society of Gastroenterology, the French Association of Surgery, the French Society of Radiology, the French-Speaking Society of Clinical Nutrition and Metabolism, and the French Society of Digestive Endoscopy ^a^ [13]	Probably not recommended to administer prophylactic anti-infective therapy in the absence of documented infection.	Weak ^a^; Strong agreement (≥70%)	NR
		Probably recommended to administer probabilistic anti-infective therapy targeting resistant enterobacteria, *Enterococcus faecium*, *Pseudomonas aeruginosa*, and yeast, to reduce morbidity and mortality in patients with infected necrosis.	Weak ^a^; Strong agreement (≥70%)	NR
2020	American Gastroenterological Association Institute Clinical Practice Update [26]	Antimicrobial therapy is best indicated for culture-proven infection in pancreatic necrosis or strong suspicion of infection (i.e., gas in collection, bacteraemia, sepsis, or clinical deterioration).	NR	NR
		Routine use of PABs to prevent infection of sterile necrosis is not recommended.	NR	NR
2019	World Society of Emergency Surgery ^a^ [10]	Routine PABs not recommended in any patients with AP.	Strong	High
		Antibiotics are always recommended to treat infected severe AP, but diagnosis is challenging.	Weak	High
		In patients with infected necrosis, empiric antibiotics should include both aerobic and anaerobic Gram-negative and Gram-positive microorganisms, and should be able to penetrate pancreatic necrosis.	Strong	Moderate
2018	American Gastroenterological Association Institute Guideline ^a^ [11]	PABs should not be used in predicted severe AP and necrotising AP.	Conditional ^c^	Low
2015	Japanese Guidelines ^a^ [12]	PABs not necessary in mild AP due to low incidence and mortality rates from infectious complications.	Strong	High
		PABs may improve prognosis in severe AP if carried out in early phases of pancreatitis (<72 h from onset).	Weak	Moderate
2013	International Association of Pancreatology/American Pancreatic Association ^a^ [8]	PABs not recommended to prevent infectious complications in AP.	Strong	Moderate
2006	American College of Gastroenterology [14]	PABs not recommended to prevent infectious complications in necrotising pancreatitis.	NR	III ^d^
2005	United Kingdom Working Party on Acute Pancreatitis [15]	No consensus on the use of PABs against infection for necrotising pancreatitis. If prophylaxis is used, it should be given a maximum of 14 days.	Grade B ^e^	NR

^a^ Defined using the definitions used in the GRADE system. ^b^ Modified definitions of the GRADE system, where A = predicted outcome was unlikely to change with future research; B = future research may have an important influence on the outcome prediction, and prediction may change; C = future research to have significant impact on confidence of prediction, with results likely to change. ^c^ Different choices will be appropriate for different patients. Decision aids may be useful in helping individuals in making decisions consistent with their values and preferences. Clinicians should expect to spend more time with patients when working toward a decision. ^d^ Level III evidence—evidence from published well-designed trials without randomisation, single-group pre/post, cohort, time series, or matched case–control studies. ^e^ Grade of recommendation was not defined in the study. AP: acute pancreatitis; GRADE: grading of recommendations, assessment, development, and evaluations); NR: not reported; PABs: prophylactic antibiotics.

**Table 3:** Summary of existing meta-analyses on the use of prophylactic antibiotics in acute pancreatitis.

Number	First Author, Year	Search Dates	Inclusion Criteria	Overall Number of Studies/Number of Patients (Prophylactic Antibiotics/No Prophylactic Antibiotics)	Type of Antibiotic	Timing of Antibiotics	Mortality/Infective Complications	Other Outcomes
1	Poropat, 2022 [29]	Inception—February 2021	RCT only AP of any severity	21 RCTs/1383 (703/680)	Ampicillin, ceftazidime, ciprofloxacin, ofloxacin, metronidazole, imipenem, imipenem–cilastin, meropenem, colistin sulfate + amphotericin + norfloxacin + cefotaxime	NR	No difference in mortality (RR: 0.85, 95% CI: 0.66–1.10) Reduced sepsis (RR: 0.43, 95% CI: 0.25–0.73) Reduced UTI (RR: 0.46, 95% CI: 0.25–0.86) No difference in pneumonia (RR: 0.73, 95% CI: 0.49–1.09), IPN (0.81, 95% CI: 0.63–1.04), bacteraemia (RR: 0.92, 95% CI: 0.33–2.58)	Reduced LOS (MD: −6.65 days, 95% CI: −8.86 to −4.43) No difference in organ failure (RR: 0.82, 95% CI: 0.65–1.03, acute renal failure (RR: 0.78, 95% CI: 0.46–1.35), acute respiratory failure (RR: 0.77, 95% CI: 0.50–1.18) No difference in need for surgical interventions (RR: 0.79, 95% CI: 0.58–1.07)
2	Guo, 2022 [17]	Inception—February 2021	Observational study/RCT Severe AP	7 studies (5 RCTs, 2 retrospective)/3846 (2757/1107)	Carbapenem	48–120 h from symptom onset, NR in 2 studies	No difference in mortality (OR: 0.69, 95% CI: 0.41–1.16) Reduced overall infections (OR: 0.27, 95% CI: 0.08–0.87) No difference in IPN (OR: 0.74, 95% CI: 0.44–1.23), EPI (OR 0.64, 95% CI: 0.15–2.75), pulmonary infection (OR: 1.23, 95% CI: 0.44–3.44), blood infection (OR: 0.60, 95% CI: 0.20–1.76), UTI (OR: 0.97, 95% CI: 0.30–3.16)	No difference in ARDS (OR: 0.80, 95% CI: 0.33, 1.91), organ failure (OR: 0.63, 95% CI: 0.32–1.24), dialysis (OR: 2.34, 95% CI: 0.12–45.21) No difference in need for surgical intervention (OR: 0.97, 95% CI: 0.53–1.79) Reduced overall local and/or systemic complications (OR: 0.48, 95% CI: 0.28–0.84)
3	Ding, 2020 [30]	Inception—June 2019	RCTs only AP of any severity	11 RCTs/747 (376/371)	Cefuroxime, ciprofloxacin, ofloxacin, imipenem, meropenem	48–120 h from symptom onset, NR in 3 studies	No difference in mortality (OR: 0.71, 95% CI: 0.44–1.15) No difference in IPN (OR: 0.74, 95% CI: 0.50–1.09) Reduced EPI (OR: 0.59, 95% CI: 0.42–0.84), UTI (OR: 0.44, 95% CI: 0.22–0.89) No difference in pneumonia (OR: 0.61, 95% CI: 0.32–1.14), positive blood culture (OR: 0.61, 95% CI: 0.32–1.14)	NR
4	Ukai, 2015 [16]	1993–2009 ^a^	RCTs only Severe AP or necrotising AP	6 RCTs/397 (202/195)	Cefuroxime, ciprofloxacin, imipenem	Within 72 h after onset/48 h after admission	Reduced mortality (OR: 0.48, 95% CI: 0.25–0.94) Reduced IPN (OR: 0.55, 95% CI: 0.33–0.92) No difference in EPI (OR: 0.62, 95% CI: 0.22–1.75)	No difference in surgical intervention (OR: 0.78, 95% CI: 0.48–1.26)
5	Lim, 2015 [34]	Inception—October 2013	Observational studies/RCTs Both severe and necrotising AP	11 studies (9 RCTs, 2 cohort)/864 (451/413)	Cefuroxime, ceftazidime, metronidazole, ciprofloxacin, ofloxacin, imipenem, meropenem	Within 48 to 120 h from onset, NR in 3 studies	Reduced mortality (RR: 0.66, 95% CI: 0.46–0.95) No difference in IPN (RR: 0.74, 95% CI: 0.51–1.07) No difference in EPI in overall cohort (RR: 0.69, 95% CI: 0.47–1.02, *p* = 0.06)	No difference in surgical intervention (RR: 0.84, 95% CI: 0.61–1.16)
6	Wittau, 2011 [31]	1966–December 2009	RCTs only SAP only	14 RCTs/841 (420/421)	Cefuroxime, metronidazole, ciprofloxacin, ofloxacin, imipenem, meropenem	NR	No difference in mortality (RR: 0.74, 95% CI: 0.50–1.07) No difference in IPN (RR: 0.78, 95% CI: 0.60–1.02) No difference in EPI (RR: 0.70, 95% CI: 0.46–1.06)	No difference in surgical intervention (RR: 0.93, 0.72–1.20)
7	Yao, 2010 [32]	January 1990–March 2010	RCTs only ANP	9 RCTs/564 (287/277)	Cefuroxime, metronidazole, ciprofloxacin, imipenem, meropenem	NR	No difference in mortality (RR: 0.69, 95% CI: 0.44–1.08) Reduced IPN (RR: 0.73, 95% CI: 0.84–0.98) No difference in EPI (RR: 0.67, 95% CI: 0.43–1.04)	No difference in surgical intervention (RR: 0.81, 95% CI: 0.59–1.10)
8	Villatoro, 2010 [33]	January 1966–November 2008	RCTs only SAP with necrosis only	7 RCTs/404 (203/201)	Cefuroxime, ciprofloxacin, ofloxacin, metronidazole, imipenem, meropenem	NR	No difference in mortality (RR: 0.60, 95% CI: 0.34–1.05) No difference in IPN (RR: 0.82, 95% CI: 0.57–1.26), EPI (RR: 0.62, 95% CI: 0.36–1.06) or any infections (RR: 0.69, 95% CI: 0.44–1.09)	No difference in surgical intervention (RR: 0.90, 95% CI: 0.62–1.31)

^a^ Search dates are not specified, only the dates of articles published are described. ANP: acute necrotising pancreatitis; AP: acute pancreatitis; ARDS: acute respiratory distress syndrome; CI: confidence interval; EPI: extrapancreatic infection; IPN: infected pancreatic necrosis; LOS: length of stay; OR: odds ratio; RCT: randomised controlled trial; RR: relative risk; SAP: severe acute pancreatitis; UTI: urinary tract infection.

**Table 4:** Summary of meta-analyses that reported the outcome variables of interest and stratified based on study type and inclusion criteria.

	No. of Meta-Analyses with Significantly Better Results in Prophylactic Antibiotics Group *	Total No. of Meta-Analyses That Included *		Total No. of Meta-Analyses with Positive Results That Included ^^^	
		Randomised Controlled Trials Only	Severe Acute Pancreatitis and/or Acute Necrotising Pancreatitis Only	Randomised Controlled Trials Only	Severe Acute Pancreatitis and/or Acute Necrotising Pancreatitis Only
Primary outcome					
Mortality	2/8 (25.0%)	6/8 (75.0%)	6/8 (75.0%)	1 /2 (50.0%)	2/2 (100%)
Infected pancreatic necrosis	2/8 (25.0%)	6/8 (75.0%)	6/8 (75.0%)	2/2 (100%)	2/2 (100%)
Overall infection/sepsis	2/3 (66.7%)	2/3 (66.7%)	2/3 (66.7%)	1/2 (50.0%)	1/2 (50.0%)
Extrapancreatic infection	1/7 (14.3%)	5/7 (71.4%)	6/7 (85.7%)	1/1 (100%)	0/1 (0)
Pulmonary infection	0/3 (0)	2/3 (66.7%)	1/3 (33.3%)	N/A	N/A
Urinary tract infection	1/2 (50.0%)	1/2 (50.0%)	1/2 (50.0%)	1/1 (100%)	0 (0)
Secondary outcomes					
Length of stay	1/1 (100%)	1/1 (100%)	0/1 (0)	1/1 (100%)	0 (0)
Organ failure	0/2 (0)	1/2 (50.0%)	1/2 (50.0%)	N/A	N/A
Acute renal failure	0/1 (0)	1/1 (100%)	0/1 (0)	N/A	N/A
Acute respiratory failure	0/1 (0)	1/1 (100%)	0/1 (0)	N/A	N/A
Need for surgical intervention	0/7 (0)	5/7 (71.4%)	6/7 (85.7%)	N/A	N/A

* Expressed over the total number of studies that reported these outcomes. ^^^ Expressed over the number of studies that reported significantly better results in the prophylactic antibiotics group.
